# CD73, a significant protein in liver diseases

**DOI:** 10.3389/fmed.2023.1147782

**Published:** 2023-04-12

**Authors:** Huilian Shi, Heng Dai, Qianqian Sun, Siliang Wang, Yuanyuan Chen

**Affiliations:** ^1^Department of Infectious Diseases, Affiliated Hospital of Nanjing University of Chinese Medicine, Nanjing, Jiangsu, China; ^2^Affiliated Hospital of Nanjing University of Chinese Medicine, Nanjing, Jiangsu, China; ^3^Department of Pharmacy, Nanjing Drum Tower Hospital, The Affiliated Hospital of Nanjing University Medical School, Nanjing, China; ^4^Department of Biochemistry and Molecular Biology, Nanjing Medical University, Nanjing, Jiangsu, China

**Keywords:** CD73, adenosine pathway, liver diseases, HBV, HCV

## Abstract

Purine adenosine pathway exists widely in the body metabolism, and is involved in regulating various physiological processes. It is one of the important pathways of environmental regulation in human body. CD73 is essentially a protease that catalyzes further dephosphorylation of extracellular adenine nucleotides, hydrolyzing extracellular AMP to adenosine and phosphate. CD73 is an important part of the adenosine signaling pathway. Studies have shown that CD73-mediated adenosine pathway can convert the inflammatory ATP into the immunosuppressant adenosine. This paper aims to summarize the relevant effects of CD73 in the occurrence, development and prognosis of liver diseases such as viral hepatitis, highlight the important role of CD73 in liver diseases, especially in viral hepatitis such as HBV and HCV, and explore new clinical ideas for future treatment targets of liver diseases.

## 1. Introduction

As of late, the frequency of liver diseases has expanded decisively year by year, and liver diseases have turned into a significant clinical issue on the planet. It is estimated that 1.5 billion individuals worldwide suffer from the ill effects of persistent chronic liver diseases (1). The Asia-Pacific region accounted for 62.6 percent of liver disease deaths globally in 2015, as per the Lancet Commission on Gastroenterology and Hepatology. Hepatitis virus infection, particularly the transmission of hepatitis B virus (HBV), is the essential driver of death in more than half part of patients with cirrhosis (2). In 2017, the World Health Organization announced that there are 324 million people with viral hepatitis around the world, and 1.34 million individuals pass away from the infection every year. In this way, it is of extraordinary importance to investigate the inward physiological system and biomolecular focuses of liver diseases, especially for viral hepatitis, to work on the better quality of human life.

Purinergic signaling was first proposed and laid out by the notable scientist Jeffrey Bernstock (3). It is basically composed of adenosine triphosphate (ATP), adenosine diphosphate (ADP), adenosine monophosphate (AMP), various adenosine kinases and receptors. It is ubiquitous in the entire body metabolism (4) and has been widely studied and discussed in various diseases. As a major adenosine kinase, CD73 is a critical component of the extracellular adenosine pathway and can be expressed and labeled on an assortment of cell surfaces. CD73 is generally present in liver tissues and profoundly expressed in liver pathology, which indicates that CD73 assumes an important part in liver diseases. This paper aims to summarize the relationship between CD73 and liver physiological and pathological phenomena, highlight the significance of CD73 in liver diseases, in order to reveal potential biological links and provide new ideas for clinical treatment strategy innovation.

## 2. Manuscript formatting

### 2.1. Biological characteristics of CD73

CD73, complete name extracellular-5′-nucleotide enzyme, is a 70-kD glycoylphosphatidylinositol (GPI), a multifunctional transmembrane glycoprotein anchored to the surface of cell membranes by 523 amino acids encoded by NT5E gene (located at 6q14-21). As an exonucleotide enzyme, CD73 has enzyme-induced and non-enzymatic functions in cells, which can catalyze further dephosphorylation of extracellular adenine nucleotides and hydrolyze extracellular AMP into adenosine and phosphate (5), acting an important role in purinergic signaling pathways (Table 1).

**Table 1:** English abbreviation comparison table.

Full name	Abbreviation	Full name	Abbreviation
Ecto-50-Nucleotidase	CD73	Primary biliary cirrhosis	PBC
Adenosine triphosphate	ATP	Primary sclerotic cholangitis	PSC
Adenosine diphosphate	ADP	Non-alcoholic fatty liver disease	NAFLD
Adenosine monophosphate	AMP	Bile duct ligation	BDL
Cyclic adenosine monophosphate	cAMP	T1 helper cells	Th1
Adenosine	ADO	T17 helper cells	Th17
T regulatory cell	Treg	T helper cells	Th
Hepatitis B virus	HBV	Aatural killer T cells	NKT
Hepatitis C virus	HCV	Hepatic stellate cells	HSC
Hepatitis E virus	HEV	Thioacetamide	TAA
Chronic hepatitis B	CHB	Alcoholic liver fibrosis	ALF
Chronic hepatitis C	CHC	Tumor microenvironment	TME
T Effector cell	Teff	Myeloid derived suppressor cells	MDSC
Autoimmune liver disease	AILD	Hepatocellular carcinoma	HCC
Autoimmune hepatitis	AIH	Vascular endothelial growth factor	VEGF

**Figure 1:**
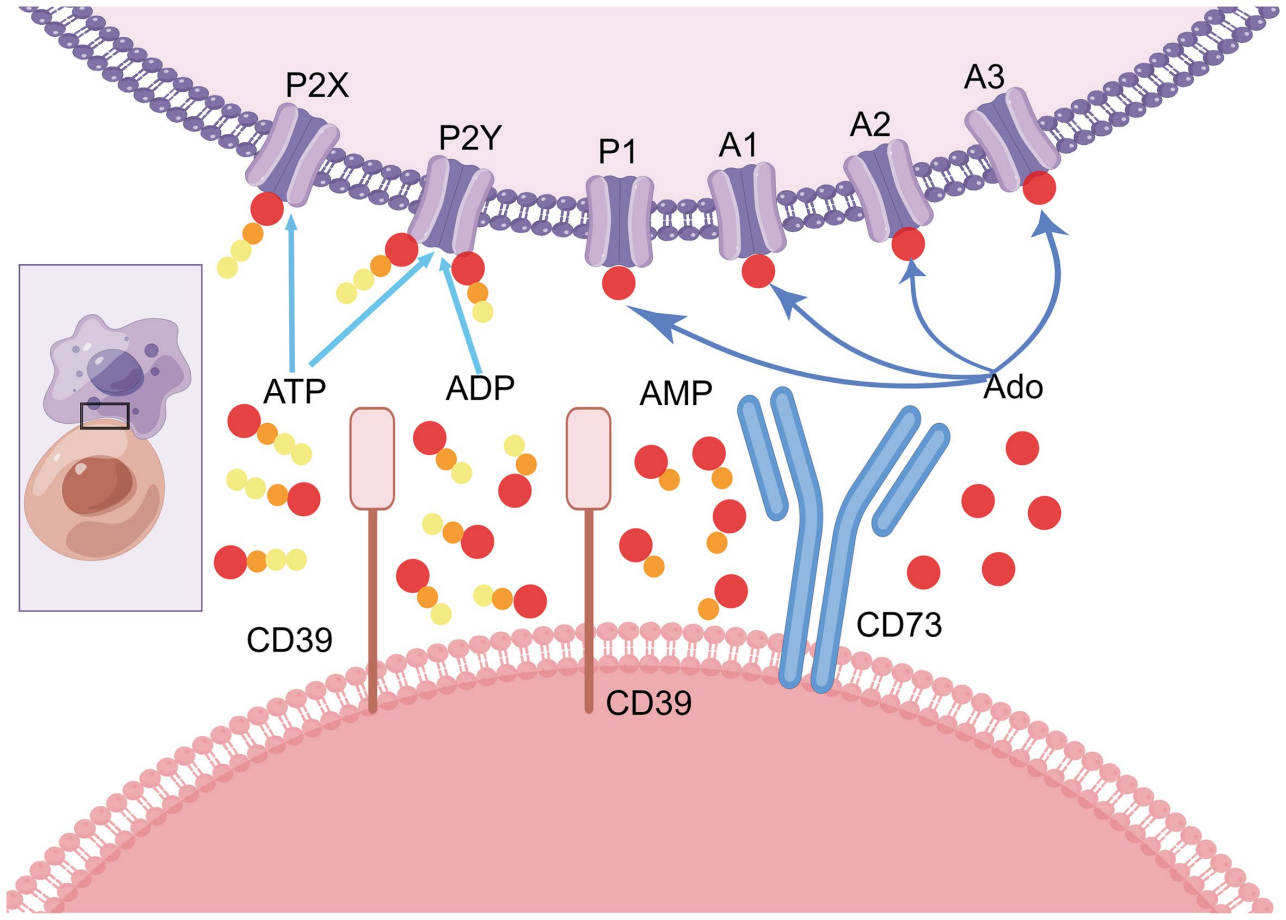
Adenosine signaling mediated by CD73. Affected by various environmental factors, extracellular pro-inflammatory risk factor ATP can bind to receptors such as P2X and P2Y to induce intense inflammation in the human body. Under the guidance of extracellular inflammation and energy balance mechanism, excessive pro-inflammatory ATP is gradually dephosphorylated to form AMP with the help of cell surface enzymes such as CD39, ALP and NTPases. AMP is further dephosphorylated by CD73 to produce adenosine which has immunosuppressive effects. Adenosine can tie to various adenosine receptors on cell surface like P1, A1, A2 and A3, and participate in various physiological and pathological reactions of the body, playing different functions and roles. CD73 is the absolute most significant hydrolytic protease for the conversion of AMP to adenosine. It is a critical step before adenosine binds to adenosine receptors and plays a vital role in the regulation and conduction of purine signaling pathways (by figdraw, export ID: YIYRA26629).

As an important downstream piece of the extracellular adenosine pathway, CD73 can convert AMP from upstream extracellular ATP and ADP hydrolyzed by cell surface enzymes such as CD39, ALP and NTPases into adenosine (ADO). Including the binding of four G-protein-coupled receptors (GPCR) subtypes A1, A2A, A2B, and A3 adenosine receptors (ARs) (6), activating a multi-step coordinated cascade of intracellular signaling pathways (7), and regulating the aggregation and dispersion of proteins all through cells by changing the CD73 enzyme activity in purine metabolism (8). Jointly play anti-inflammatory, dilated blood vessels and many other functions (Figure 1).

CD73 is expressed in a grouping of cell types, including leukocytes, myofibroblasts, endothelial cells and epithelial cells, and so on (9), particularly in tumor, immune and other related cells (10), such as macrophages, myofibroblasts, dendritic cells and NK cells (11), etc. CD73 is also expressed in neutrophils to a certain extent, which can propel liver regeneration and regulate inflammation (12). In ongoing years, the mechanism of adenosine generating enzyme CD73 has been preliminarily researched in liver diseases, including viral hepatitis, hepatic steatosis, hepatic fibrosis and hepatocellular carcinoma. Continuous examinations have shown that under the influence of multiple factors, CD73-mediated adenosine metabolism is immovably connected with the liver and dynamically regulates various pathological manifestations such as liver steatosis, inflammation, fibrosis and primary tumor (13), prompting the occurrence of all kinds of liver diseases.

### 2.2. CD73 and viral hepatitis

As a universally prevalent chronic liver disease, viral hepatitis is a typical common infectious disease caused by a variety of hepatotropic viruses. It can cause intense and persistent liver inflammation in people, and then develop into cirrhosis and liver cancer, which seriously endangers human wellbeing globally. Purinergic signaling is firmly related to the generation and progression of liver inflammation (14). As a perilous signal that promotes inflammation, ATP has an invigorating unstable quality (15). On the contrary, ADO has anti-inflammatory effects on immune cells and can safeguard tissue integrity (16). As a key component of the adenosine pathway, CD73 is one of the cell surface compounds that decompose extracellular ATP into ADO, promoting the transformation of the body from the pro-inflammatory environment stimulated by ATP to the anti-inflammatory environment directed by adenosine (13).

After the liver is hit by the virus infection and produces inflammation, the level of ATP which is a dangerous signal of extracellular inflammation increases significantly (17), stimulates and induces neutrophils to aggregate and produce chemokines (18), and recruit immune cells with high expression of CD73 (19), deplete extracellular ATP to produce adenosine, inhibit the activation of immune cells, and control the inflammatory response (10). Simultaneously, activation of adenosine receptors can increment intracellular AMP concentration, structure a partition hindrance, block intercellular substance trade, and avoid the abnormal accumulation of immune cells to stimulate inflammation (20). CD73-intervened adenosine pathway takes immune cells such as B cells and Tregs as the principal carriers to promote adenosine production and suppress immunity, which plays an important role in the pathogenic process of several common hepatitis viruses and is considered as a new possible therapeutic target for liver viral inflammatory diseases (21).

In patients with chronic hepatitis B (CHB), when liver inflammation occurs, CD73 on the surface of B cells is diminished, the amount of extracellular adenosine production is decreased, and the activation of B cells is enhanced, prompting the progression of inflammation (22). Blocking CD73 activity in CHB patients can lead to impaired IgG conversion of B cells, temporarily slowing down humoral immune inflammation, but further aggravating later inflammation in the long term (23). As of now, there is no clear report on the internal mechanism of CD73 increase in Treg cells of patients with hepatitis B, but some studies have shown that CD73 is an important regulator of Treg cells to restrain intracellular environmental inflammation, and the author conjectures that it may be related to the independent expression of CD73 in Treg cells under inflammatory environment and the immunosuppressant effect mediated by adenosine (24). In addition, studies have found that the expression level of CD73 decreases with the increase of HBV-DNA load and liver inflammatory response. In patients with complete antiviral response, the amount of CD73 can be gradually recovered with the transformation of serum HBeAg or the reduction of HBsAg, yet after effective antiviral treatment, the expression of CD73 does not increase (22).

In addition to hepatitis B, TOX+ HCV-specific CD8 + T cells in patients infected with hepatitis C virus (HCV) express a mass of CD73 characteristic memory phenotype (25), especially in activated Treg cells (26), playing an important role in inflammation in patients with chronic hepatitis C (CHC). ZhiqinLi believes that during antiviral treatment, the overall number of Treg cells expressing higher levels of CD73 in CHC patients show a downward trend, which also explain the reason why NatashaT. Snider found in the study that the liver CD73mRNA level of CHC patients is significantly reduced (9). This phenomenon is more obvious in liver fibrosis caused by HCV (10).

In patients with chronic hepatitis E, the expression of CD73 in different cells is also significantly different. For example, studies found that the expression of CD73 on the surface of Tregs and effector T cells (Teff) in patients infected with hepatitis E virus (HEV) was increased. Moreover, the inhibitory ability of Treg cells in patients with acute hepatitis E is obviously higher than that in recovered individuals (27), which might be influenced by CD73-mediated adenosine pathway during the course of the disease. However, the activity and function of CD73 on B cell surface under HEV invasion need to be further investigated.

As for hepatitis A and hepatitis D, few similar articles have mentioned the effect of CD73 on hepatitis A. Undeniably, we found that the infection of hepatitis D depends on the replication of hepatitis B virus itself, and adenosine receptors are the necessary proteins for human hepatocytes to infect two viruses (28). However, no studies have discussed the effect of CD73 on hepatitis D through adenosine pathway, so the intervention of CD73 on the pathogenesis of hepatitis A and hepatitis D needs to be further explored.

### 2.3. CD73 and other liver diseases

With the improvement of modern living standards, coupled with unhealthy diet and hygiene habits, the incidence of various liver diseases increases year by year (29). Various chronic liver diseases persist and are prone to progress to irreversible end-stage liver disease, affecting normal metabolism of the body and eventually leading to death (30).

Studies have proven that CD73 is expressed at a high level in liver tissues (31), which is mainly distributed in the apical membrane of hepatocytes and endothelial cells of hepatic sinuses and bile ducts, and is expressed in bands in pericentral hepatocytes near the central vein of the liver (32). In CD73-deficient hepatocytes, AMP-dependent protein kinases are affected, resulting in the destruction of liver homeostasis and unexpected liver injury (33). What’s more, the expression of CD73 is highly regulated in chronic liver diseases (9). In terms of current research progress, CD73 can inhibit the progression of viral hepatitis to a certain extent, promote the formation of fatty liver, delay the progression of steatohepatitis, and promote the progression of liver fibrosis and liver cancer through the adenosine pathway.

#### 2.3.1. CD73 and autoimmune liver disease

Autoimmune liver disease (AILD) is a chronic, progressive immune-related liver disease caused by the activation of the body’s immune system function due to various unknown reasons. As the final rate-limiting enzyme of adenosine production, CD73 mediates purine signaling pathway, plays an important part in regulating adenosine and immune diseases (25). Tregs act an irreplaceable role in AILD (34). Studies indicate that autoimmune hepatitis (AIH) and primary biliary cirrhosis (PBC) are closely related to adenosine pathway mediated by CD73 on Treg surface.

AIH, as a chronic liver disease caused by abnormal activation of immune cells leading to interfacial hepatitis (35), is a common clinical AILD type (36). CD73-mediated adenosine pathway is immunosuppressive toward AIH, and Treg impairment and Teff subgroup activation are typical manifestations of AIH pathogenesis (13). CD73 around the surface of normal Treg cells mediates the production of immune-suppressing adenosine (24), blocks cell communication, inhibits overimmunity, and simultaneously upregates CD73 expression (10). However, the level of CD73 on the damaged Treg surface was down-regulated (37), the secretion of TGF-β and other anti-inflammatory factors was reduced (38), and the immunosuppressive function of Treg was defective (39), leading to the occurrence of AIH. On the other hand, IL-6 and TGF-β can induce the expression of CD39 and CD73 in helper T17 cells (Th17), stimulate the production of adenosine, inhibit the transformation of naive T cells into Th (40), along with reducing the production of pro-inflammatory cytokines (41), which can be charactered as immune deficiency. On the contrary, low level of CD73 stimulates the production of pro-inflammatory factors in liver cells, and the immune effect of Th17 cells continue (42), leading to the continuous progression of chronic liver inflammation.

PBC is an immune-related liver disease characterized by diffuse destruction of small bile ducts in the liver (43). The literature indicates that there may be significant individual differences in the mechanism of PBC effect (44). There are few studies on the progression of CD73 in PBC disease. Due to immune deficiency, CD73 expression in dnRIITreg from PBC mice is significantly reduced compared with WTTreg. Studies found that there are certain differences in the expression profiles of CD39 and CD73 on Tregs, which can form energy circle outside immune cells and mediate a series of immune responses, which may be close to the pathogenesis of PBC, and its internal mechanism is worthy of further lucubrating (37).

Unlike PBC, the lesions of primary sclerosing cholangitis (PSC) are mainly in the bold ducts inside and outside the liver. At present, relevant studies on the pathogenesis of CD73 in PSC are still lacking, but previous studies have shown that the loss of CD39 in the adenosine pathway can stimulate the increase of intestinal endocrine ATP, activate dendritic cells and CD8 + T cells and transport them to the liver. Damage to biliary epithelial cells induces PSC (45), which also proves that adenosine pathway is closely connected to the progression of PSC disease. Therefore, the role of CD73 in the course of PSC disease needs to be further analyzed.

#### 2.3.2. CD73 and fatty liver disease

Liver is an important organ for ethanol metabolism, and a large amount of ethanol metabolism tend to have toxic effects on the liver, resulting in hepatocyte damage (46), and then abnormal accumulation of metabolism-related fat, leading to hepatic steatosis. Studies have confirmed that CD73 activity is associated with ethanol-induced hepatic steatosis, mice lacking CD73 show less cell expansion and steatosis, significantly reducing the incidence of fatty liver (47). Wang, Ping et al. hypothesized that CD73-deficient mice may reduce adenosine-mediated extracellular matrix deposition through hepatic stellate cells, thereby protecting mice from ethanol induced fatty liver (10). Under chronic alcohol stimulation, AMP is released in the liver (48) and phosphorylated to adenosine catalyzed by high expression of CD73 on the cell surface, adenosine A1 and A2B receptors are activated and promote lipid depositional degeneration (47). At the same time, ethanol absorption also reduces the influx of nucleoside transporter (49), inhibits intracellular adenosine uptake and increases extracellular adenosine concentration (50), promoting the progression of fatty liver. Therefore, blocking the expression of CD73, A1 or A2B receptors in the liver can effectively reduce the accumulation of liver lipids caused by alcohol and delay the course of fatty liver disease (26).

At the same time, non-alcoholic fatty liver disease (NAFLD) is another important cause of hepatic steatosis in modern society (51). Non-alcoholic steatohepatitis (NASH), as a typical inflammatory disease of NAFLD, is closely related to the adenosine pathway. As a hydrolytic product of CD73, adenosine can perform cell protective and immunosuppressive functions through P1 receptors, thus terminating liver inflammation and promoting liver regeneration. In addition, CD73 can block the TLR4/MyD88/NF-κB signaling pathway (52), reduce the secretion of IL-6 and IL-1β, and delay the inflammatory process. In liver biopsies of NAFLD patients, CD73mRNA levels were significantly reduced (9), so CD73 knockout mice rarely developed fatty liver disease, or even progressed to steatohepatitis (13). However, under chronic inflammatory stimulation, damaged inflammatory liver cells can induce the approach of extracellular immune cells with high expression of CD39 and CD73, clear extracellular ATP, generate adenosine negative feedback to regulate endothelial cells and immune cells, inhibit white blood cell recruitment, and reduce the inflammatory response. The study on the connection between CD73-related adenosine metabolism pathway and hepatic fatty lesions is worth further exploration.

#### 2.3.3. CD73 and liver cirrhosis

Hepatic fibrosis is a reaction of repeated prolongation of various chronic liver lesions, causing liver self-limiting healing (53), which can lead to the formation of cirrhosis. CD73, as the final rate-limiting enzyme produced by adenosine (25), has an important place in the process of liver fibrosis. CD73 is weakly expressed in normal hepatic stellate cells and portal vein fibroblasts, while its activity is enhanced in hepatic fibrosis (54) and significantly increased in cirrhosis (55). Among them, hepatic stellate cell (HSC) activation is a pivotal feature of hepatic fibrosis, CD73 and HSC activation interact with each other to jointly promote the process of hepatic fibrosis (56). For example, the CD73-adenosine-A1R axis regulates HSC activation and apoptosis through the PLC-IP3-Ca2+/DAG-PKC signaling pathway (57). CD73-deficient mice are resistant to the development of liver fibrosis (58) and protect liver cells from the risk of CCl4 and thioacetamide (TAA) inducing liver fibrosis (59). After CD73 deletion, adenosine production is reduced, resulting in the suppression of HSC activation and proliferation mediated by p2 receptor and decreased collagen expression, inhibiting the production of liver fibrosis. Meanwhile, inhibition of CD73 can promote HSC apoptosis and alleviate alcohol-induced liver fibrosis (52). Similarly, A2A adenosine receptor deficient mice were also protected from the effects of liver fibrosis by blocking the adenosine pathway (60). Just the opposite, after alcohol intake, CD73 is activated in acetaldehyde induced HSC, and the expressions of pro-fibrotic cytokines TGF-β, α-SMA and type I and III collagen are increased (61), promoting the generation of liver fibrosis. Activated HSC can up-regulate CD73 expression through specific SP1 and SMAD promoter elements (58). These studies have verified the intrinsic influence of CD73 expression and HSC activation and their co-promoting effect on liver fibrogenesis. Therefore, blocking CD73 expression may be an important approach for the treatment of hepatic fibrosis.

#### 2.3.4. CD73 and hepatocellular carcinoma

Hepatocellular carcinoma (HCC) is one of the most common causes of cancer-related death and is one of the most frequent malignant lesions of digestive tract in the world (62). A large number of literatures have elaborated that CD73 is an important regulatory protein in the progression of various malignant tumors and is highly expressed in cancer tissues (63). CD73 was significantly increased in HCC patients and negatively correlated with overall survival (64). Studies have stated that CD73 is highly expressed in about 50% of HCC samples (55), which promotes the progression and metastasis of tumors, and can be used as a reference indicator for poor prognosis of HCC clinical outcomes (65). Recent studies claimed that tumor microenvironment (TME), as the basis for tumor survival, provides the driving force for tumor proliferation and metastasis (64). Purinergic signaling pathway is the main immunosuppressive mechanism of TME (66). As one of the core enzymes of adenosine pathway, CD73 is expressed on the surface of tumor endothelial cells, regulatory T cells (Treg), NK cells, medullogenic suppressor cells (MDSC), tumor-associated macrophages and other cells of TME. Regulated by epidermal growth factor receptor (HER) and other molecules (67), TME can induce immune escape to improve intracellular AMP level and start downstream signaling pathways, such as promoting proliferation induction of Tregs (68), blocking Teff aggregation and invasion (69), reducing NK cytotoxicity (70), and stimulating MDSC and macrophage polarization (71). Inhibit the production of cytokines, reduce the antigen-presenting effect of tumor (72), and inhibit anti-tumor response. CD73 has also been verified to motivate the increase of tumor vascular endothelial growth factor (VEGF), promote angiogenesis (73) and help tumor cells survive (74). Additionally, CD73 may also be in relation to inflammatory cancer signal transduction in liver cancer. Through A1R, A2AR, A2BR, and A3 adenosine receptor signaling pathways, CD73 stimulates inflammation, provides a suitable pro-inflammatory environment for tumor cells to survive, and plays an important role in the occurrence, development and metastasis of hepatocellular carcinoma. As mentioned above, inflammatory cells generally have low expression of CD73, while HCC cells with high expression of CD73 release a large number of inflammatory factors, which may be due to the fact that adenosine pathway is not the main pathway for the generation of inflammation in tumor cells. The influence of CD73 on the relationship between inflammatory and carcinoma transformation needs to be further explored (Tables 2, 3).

**Table 2:** List of related mechanisms of CD73 in liver diseases.

Type of liver disease	Correlation with CD73 and relevant mechanism of action	References
Viral hepatitis	The virus invades the liver, significantly increases ATP levels, stimulates and induces neutrophils to produce chemokines, recruits immune cells with high expression of CD73, consumes extracellular ATP to produce adenosine, inhibits immune cell activation, and controls inflammation	(10, 17–19)
	Activation of adenosine receptors increases intracellular AMP concentration, forming a barrier that blocks the exchange of substances between cells and prevents the abnormal accumulation of immune cells from stimulating inflammation	(20)
	In CHB patients, CD73 and extracellular adenosine production on the surface of B cells at the site of liver inflammation were decreased, and the activation of B cells was enhanced through IgG conversion, inducing inflammation progression	(22, 23)
	CD73 expression decreased with the increase of HBV-DNA and liver inflammatory response. In patients with complete antiviral response, CD73 levels recovered gradually with serum HBeAg conversion or HBsAg reduction, and CD73 did not increase after effective antiviral therapy	(22)
	Activated Treg cells in CHC patients showed high expression of CD73, but the number of Treg cells decreased, resulting in a significant decrease in overall CD73 levels	(9, 25)
	The expression of CD73 on the surface of Treg and Teff cells was increased in patients with hepatitis E, but the inhibition ability of Treg cells was also increased	(26)
Autoimmune hepatitis	The level of CD73 on the surface of AIH-damaged Treg cells was down-regulated, and the secretion of TGF-β and other anti-inflammatory factors was reduced, leading to the deficiency of immunosuppressive function of Treg	(36–38)
	IL-6 and TGF-β induced decreased CD73 expression in Th17 cells, blocked adenosine production, stimulated more naive T cells to transform to Th, produced pro-inflammatory cytokines, and increased inflammation	(39, 40)
	Low expression of CD73 stimulates the production of pro-inflammatory factors in hepatocytes, and low level of A2A receptor on Th17 cell surface activates, and the immune effect continues, leading to chronic liver inflammation	(41)
Primary biliary cirrhosis	Due to immune deficiency, the expression profiles of CD39 and CD73 on Treg are different to some extent, which can form “purinergic halo” outside immune cells and mediate a series of immune responses, which may be closely related to the pathogenesis of PBC	(36, 43)
Primary sclerosing cholangitis	The deletion of CD39 can stimulate the increase of intestinal endocrine ATP, activate dendritic cells and CD8 + T cells and transport them to the liver, and damage biliary epithelial cells to induce PSC. The correlation with CD73 remains to be studied	(44)
Fatty liver disease	CD73-deficient mice may protect against etho-induced fatty liver by reducing adenosine-mediated extracellular matrix deposition through hepatic stellate cells	(10)
	Under chronic alcohol stimulation, AMP is released in the liver and phosphorylated to adenosine catalyzed by high expression of CD73, which binds to cell surface adenosine A1 and A2B receptors and promotes lipid depositional degeneration	(46, 47)
	Alcohol intake reduces the influx of nucleoside transporters, inhibits intracellular adenosine uptake, and leads to increased extracellular adenosine concentration, promoting the development of fatty liver	(48, 49)
	Overexpression of CD73 can block TLR4/MyD88/NF-κB signaling pathway, reduce alcohol-induced liver injury, reduce the secretion of IL-6, IL-1β and other inflammatory cytokines, delay the inflammatory process, promote cell proliferation and inhibit cell apoptosis	(51)
	Endogenous A1A adenosine receptor activation which is mediated by CD73 alleviates ethanol-induced acute liver injury by reducing oxidative stress and lipid accumulation	(25)
Liver cirrhosis	CD73 activity is enhanced when liver fibrosis occurs and expression is significantly elevated in cirrhosis	(53, 54)
	The CD73-adenosine-A1r axis regulates HSC activation and apoptosis through the PLC-IP3-Ca2+/DAG-PKC signaling pathway	(56)
	CD73 defects are resistant to the development of liver fibrosis and protect mice from CCl4 and TAA induced liver fibrosis	(57, 58)
	Absence of CD73 and reduced adenosine production can lead to p2 receptor-mediated HSC activation and proliferation and decreased collagen expression, and inhibit the production of liver fibrosis	(10)
	Mice with deficient A2A adenosine receptors were also protected from liver fibrosis by blocking the adenosine pathway	(59)
	Extracellular ATP stimulation in acetaldehyde-induced HSC induced increased CD73 activation and the expression of TGF-β, α-SMA and type I and III collagen, promoting liver fibrosis	(60)
	Inhibition of CD73 can promote HSC apoptosis and reduce liver fibrosis induced by alcohol	(51)
	Activation of HSC can promote fibrosis progression by up-regulating CD73 expression through specific SP1 and SMAD promoter elements	(57)
	The knockdown CD73 can significantly increase cell migration and collagen I expression of HSC line, and promote fibrosis process	(60)
Hepatocellular carcinoma	The up-regulation of CD73 expression induces the gradual transformation of immune-activating and tumor-inhibiting ATP into immune-inhibiting adenosine, accumulates and activates adenosine receptors, promotes the proliferation and induction of Treg, blocks Teff aggregation, and inhibits the anti-tumor response of the immune system	(67, 68)
	The up-regulation of CD73 expression stimulates adenosine production, plays an immunosuppressive role, reduces the toxicity of NK cells, reduces the killing cleavage of tumor cells, and enables tumor survival	(69)
	High expression of CD73 induces adenosine generation and activation of adenosine receptors, increases intracellular AMP level, stimulates MDSC and macrophage polarization, inhibits cytokine production, weakens tumor antigen presentation, and inhibits anti-tumor response	(70)
	Under CD73-mediated adenosine activation, A2A receptor is highly expressed on the surface of tumor endothelial cells, stimulating VEGF production and significantly enhancing tumor angiogenesis, especially in early liver cancer	(72, 73)

**Table 3:** List of different effects of CD73 in liver diseases.

Liver diseases		The expression of CD73	Influence of CD73 on various liver diseases	Effects of CD73 blocking in different liver diseases	References
Viral hepatitis	Viral hepatitis B	The expression of CD73 on B cells surface is decreased	The amount of extracellular adenosine production is decreased, and the activation of B cells is enhanced, inducing the progression of inflammation	Blocking CD73 activity can lead to impaired IgG conversion of B cells, temporarily slowing down humoral immune inflammation, but further inducing later inflammation	(22, 23)
		The expression level of CD73 is increased in Treg cells	Treg cells independently express of CD73 under inflammatory environment and mediate the adenosine immunosuppressant effect		(24)
	Viral hepatitis C	The total expression of CD73 content of Treg cells decreases	TOX+ HCV-specific CD8 + T cells, especially activated Treg cells, increased expression of CD73, but the overall number of Treg cells decreased, and therefore overall CD73 content decreased		(25)
	Viral hepatitis E	The expression level of CD73 is increased on cell surface	CD73 expression on Treg and Teff surfaces both increases		(26)
Autoimmune liver disease	Autoimmune hepatitis (AIH)	The expression level of CD73 on Treg cells is down-regulated	Tregs are damaged and CD73 levels on the cell surface are down-regulated, the secretion of TGF-β and other anti-inflammatory factors is reduced, and the immunosuppressive function of Treg is defective		(36–38)
		Teff subgroup is activated and CD73 expression increased	IL-6 and TGF-β can induce the expression of CD73 in Th17, induce the production of adenosine, inhibit the transformation of naive T cells to Th and reduce the production of pro-inflammatory cytokines, showing the characteristics of immune deficiency		(39, 40)
	Primary biliary cirrhosis (PBC)	CD73 expression in Treg is significantly decreased	Compared with WTTreg, CD73 expression in dnRIITreg of PBC mice is significantly decreased		(36)
Fatty liver disease	Alcoholic fatty liver	CD73 is highly expressed on the surface of liver cells	Under chronic persistent alcohol stimulation, AMP is released in the liver and phosphorylated to adenosine catalyzed by CD73, which binds to cell surface adenosine A1 and A2B receptors and promotes degeneration of hepatic lipid deposition	CD73-deficient mice relatively exhibite less liver cell dilatation and steatosis, and the incidence of fatty liver is significantly reduced. Blocking the adenosine pathway in CD73 knockout mice can reduce adenosine-mediated extracellular matrix deposition through hepatic stellate cells, thereby protecting mice from ethanol-induced fatty liver. Blocking the expression of CD73, A1 or A2B receptors in liver can effectively reduce the accumulation of liver lipids caused by alcohol and delay the course of disease	(10, 25, 46, 47)
	Nonalcoholic fatty liver disease (NAFLD)	The level of CD73mRNA is significantly decreased	CD73 can improve ETOh-induced liver injury and inflammatory response. Adenosine, as a hydrolytic product of CD73, plays a role in cell protection and immunosuppression through P1 receptors, thus terminating liver inflammation. CD73 can block the TLR4/MyD88/NF-κB signaling pathway, reduce the secretion of IL-6, IL-1β and other cytokines, and delay the inflammatory process. Under chronic inflammatory stimulation, damaged inflammatory liver cells can induce the approach of immune cells with high expression of CD39 and CD73, clear extracellular ATP, generate adenosine negative feedback to regulate endothelial cells and immune cells, inhibit leukocyte recruitment, and reduce inflammatory response	The mice with CD73 knockout rarely developed fatty liver, or even progressed to steatohepatitis	(9, 10, 13, 51)
Liver cirrhosis		CD73 expression increases and the activity of CD73 is enhanced	Acetaldehyde induces activation of CD73 in HSC, and induces the increased expression of pro-fibrotic cytokines TGF-β, α-SMA and type I and III collagen, promoting the generation of liver fibrosis. Activated HSC can up-regulate CD73 expression by specific SP1 and SMAD promoter elements	CD73-deficient mice are resistant to the development of liver fibrosis and can protect liver cells from the risk of inducing cirrhosis by CCl4 and thioacetamide (TAA). After CD73 deletion, adenosine production is reduced, leading to p2 receptor-mediated HSC activation proliferation and collagen expression decrease, and inhibiting the production of liver fibrosis. Inhibition of CD73 can promote HSC apoptosis, alleviate alcohol-related liver fibrosis. A2A adenosine receptor deficient mice were also protected from liver fibrosis due to the blocking of adenosine pathway	(10, 51, 57, 59, 60)
Hepatocellular carcinoma		The expression of CD73 is significantly elevated in HCC patients	CD73 enhances intracellular AMP by inducing immune escape in TME, initiates a variety of downstream signaling pathways, and inhibits antitumor response. CD73 can induce the increase of tumor vascular endothelial growth factor (VEGF), promote angiogenesis and help tumor cells survive. CD73 may be related to inflammatory signaling in liver cancer, stimulating inflammation through adenosine receptor signaling pathway and providing a suitable pro-inflammatory environment for tumor cells to survive		(13, 67–73)

## 3. Conclusion

The latest World Health Organization figures for 2020 show that 325 million people worldwide are living with viral hepatitis B and C. Each year there are 900,000 deaths due to hepatitis B virus infection. Back in 2016, the World Health Organization set a goal of eliminating hepatitis B as a public health threat by 2030. Hepatitis virus infection is a serious harm to human health, and it is still a global public health problem worthy of attention.

The incidence of liver virus infection is related to the number of virus replication and the strength of the body’s immunity. The progression of the disease is due to the continuous replication of the virus and the poor immunity of the body, resulting in progressive damage to the liver cells, causing a series of serious consequences. At present, antiviral therapy is difficult to achieve the ideal state of completely removing virus from the body. Therefore, the focus of treatment of viral hepatitis is to regulate the immune function of the body and weaken the damage of virus metabolism to the liver.

Studies have confirmed that the CD73-mediated adenosine pathway has a profound effect on the activated immune response of B cells. The activation of B cells induced by low expression of CD73 is an essential part of the effective immune response against HBV infection and a reference idea for the future treatment of viral liver inflammation and restoration of liver immune homeostasis. On the other hand, CD73 content increases, inducing adenosine-mediated immunosuppression. Whether this is also influenced by liver pathological microenvironment such as hepatitis B viral load, liver inflammation and antiviral intervention, and the role of CD73 in cellular immunity needs to be further studied.

Similarly, under the stimulation of hepatitis C and hepatitis E virus, the expression of CD73 on the surface of activated Treg increased, but the number of Treg cells in patients with hepatitis C decreased, so the overall content of CD73 decreased, inducing immune inflammation in the liver. However, there is still a lack of experimental studies on the effect of CD73 on humoral immunity under hepatitis C and hepatitis E virus infection in the existing literature, which needs to be improved.

As an important link in the adenosine pathway, CD73 co-conducts with upstream ATP and AMP and downstream adenosine and adenosine receptors, and is involved in the occurrence and development of viral hepatitis and other liver diseases. In view of the important role of CD73 and its related metabolic pathways in liver diseases, Targeted intervention of CD73 can be one of the key breakthroughs in the future treatment of liver diseases and restoration of environmental homeostasis in the liver, which has a good application prospect. At present, there have been experimental or clinical reports on related drugs, such as metformin, which can inhibit CHB immune-related pathogenesis by regulating CD73 adenosine pathway (22). In addition, some studies have also found that CD73-related pathways in the field of traditional Chinese medicine. For example, cordycepin, as an adenosine analogue, can specifically activate adenosine receptors and improve chronic inflammation caused by liver fat accumulation in an immunosuppressive environment with high CD73 expression (75). Curcumin can inhibit the carcinogenic effect of aflatoxin on liver to a certain extent through CD73-mediated purine pathway (76), induce the differentiation of bone marrow mesenchymal stem cells, and promote liver regeneration.

Recent studies have proved that there are certain differences in the expression levels of CD73mRNA and protein in two different kinds of mice (9). This also means that the intrinsic influence of species genes on CD73 expression, including viral metabolism in the liver, needs to be further confirmed by further studies.

As an important part of the adenosine pathway, CD73 is the key to the transition from AMP to Ado, which implies partial intervention in energy metabolism and immune regulation. For CD73 itself, it exists as a protein widely spread in various cells of the body, is one of the components of human gene expression. For example, as for liver cancer, CD73 not only acts as an intermediary to help tumor cells evade immune monitoring and regulate internal environmental inflammation, but also provides nutritional support and metastasis pathway to tumor cells by promoting angiogenesis. Therefore, following studies should explore more value of CD73 affecting human metabolism and pathological changes from different new ideas and perspectives.

With the continuous development of more and more experimental studies based on protein CD73, many drugs targeting CD73 have been introduced into the clinic, which can be widely used in the field of liver disease in the future, especially providing new treatment options for patients with chronic hepatitis B and chronic hepatitis C, delaying the onset of cirrhosis and liver cancer, and hopefully improving the quality of life of patients with liver diseases.

## Author Contributions

YC conceived and guided the study. HS, HD and QS completed the main part of this work, SW is responsible for the submitting of manuscripts. All authors contributed to the article and approved the submitted version.

## Funding

This research was funded by the National Natural Science Foundation of China (NSFC) 81903974 to HS and 82070804 to YC, the Natural Science Foundation of Jiangsu Province BK20221421 to HS, and the Natural Science Foundation of Nanjing University of Chinese Medicine XZR2021022 to HS.

## Conflict of interest

The authors declare that the research was conducted in the absence of any commercial or financial relationships that could be construed as a potential conflict of interest.

The handling editor MY declared a shared parent affiliation with the author YC at the time of review.

## Publisher’s note

All claims expressed in this article are solely those of the authors and do not necessarily represent those of their affiliated organizations, or those of the publisher, the editors and the reviewers. Any product that may be evaluated in this article, or claim that may be made by its manufacturer, is not guaranteed or endorsed by the publisher.
